# Effect of acupuncture on ischemic stroke patients with hypertension: a randomized clinical trail

**DOI:** 10.3389/fneur.2026.1717706

**Published:** 2026-04-01

**Authors:** Wei Liu, Boxuan Li, Mengying Rong, Chen Li, Qi Zhao, Lili Zhang, Xiaochan Tan, Qiuxia He, Haipeng Ban, Xianggang Meng, Wenlong Gu, Xinxin Gao, Fen Ma, Mi Ou, Xuemin Shi, Yuzheng Du

**Affiliations:** 1 First Teaching Hospital of Tianjin University of Traditional Chinese Medicine, National Clinical Research Center for Chinese Medicine, Tianjin, China; 2 Tianjin University of Traditional Chinese Medicine, Tianjin, China; 3 Acupuncture Department, The First Affiliated Hospital of Zhejiang Chinese Medical University (Zhejiang Provincial Hospital of Traditional Chinese Medicine), Hangzhou, China; 4 Tianjin Academy of Traditional Chinese Medicine Affiliated Hospital, Tianjin, China; 5 Tianjin Hongshunli Street Community Health Service Center, Tianjin, China

**Keywords:** acupuncture, blood pressure, hypertension, randomized clinical trial, stroke

## Abstract

**Background:**

Limited evidence suggests that acupuncture has a short-term antihypertensive effect. The efficacy and safety of acupuncture as an adjunctive treatment for ischemic stroke patients with hypertension were uncertain.

**Objective:**

To investigate the efficacy and safety of acupuncture as adjunctive therapy in reducing stroke recurrence and improving blood pressure for stroke patients with hypertension.

**Design:**

Multicenter randomized, controlled trial.

**Methods:**

18 centers from September 2015 to January 2020. 480 stroke participants with hypertension were randomized into adjunctive acupuncture group and basic treatment group to complete a 12-week treatment. The primary outcome was the one-year recurrence of stroke and the secondary outcome in 48-week follow-up included blood pressure, the Traditional Chinese Medicine syndrome score, the Short Form 36-item Health Survey, the National Institute of Health Stroke Scale, the Barthel Index scale, the ultrasound doppler, and serum tests.

**Results:**

There were no statistical differences between groups in primary outcome (*p* = 0.63 > 0.05). After 12-week treatment, adjunctive acupuncture group: (1) showed a higher participant proportion of dipper diastolic blood pressure pattern (*p* = 0.01 < 0.05); (2) had a better score on the Short Form 36-item Health Survey (*p* < 0.001); (3) decreased the vascular resistance of Right Internal Carotid Artery (*p* = 0.03 < 0 0.05).

**Conclusion:**

Adjunctive acupuncture therapy can improve stroke patients’ circadian rhythm, carotid blood flow, and quality of life, but may not have a superior effect on reducing one-year stroke recurrence.

**Clinical trial registration:**

ClinicalTrials.gov, identifier NCT02967484.

## 1 Introduction

Hypertension, a leading cause of premature death, affects 1.39 billion people worldwide (1). It is a leading risk factor for stroke incidence and recurrence (2, 3). Systolic blood pressure (SBP) and diastolic blood pressure (DBP) were positively correlated with stroke incidence (4). Maximum SBP was associated with a 15-fold increased risk for stroke (5).

Increasing awareness and treatment adherence to hypertension has become the main goal for the prevention of stroke incidence (2). The primary suggestions for controlling hypertension are pharmacological treatment and lifestyle modification (6, 7). However, the control and compliance rates of hypertension treatment are unsatisfactory (1). The World Health Organization estimated that 50–70% of hypertension patients did not take antihypertensive drugs as prescribed (8). Fear of medication side effects is a major driver of non-adherence (9). Adverse effects led 9% of mild hypertension patients to discontinue drug therapy (10).

Acupuncture therapy, with a long history in China, has good compliance with fewer side effects (11). The effect of acupuncture on reducing blood pressure (BP) has been revealed with many potential mechanisms, including the central nervous system, renin-angiotensin-aldosterone system, vascular endothelium, and oxidative stress (12, 13). Beyond blood pressure control, acupuncture may offer additional neuroprotective benefits in stroke management. Recent evidence has shown improvements in cognitive function and modulation of autophagy-inflammatory pathways, supporting its potential as a comprehensive adjunctive therapy (14–16). Although increasing evidence has shown that acupuncture has a short-term antihypertensive effect, high-quality randomized controlled trials (RCT) are still lacking (17–20). Meanwhile, only a few clinical studies have explored the continuous antihypertensive effect of acupuncture after treatment and reached inconsistent conclusions (21, 22). A meta-analysis in the Cochrane Library found no evidence for the sustained BP-lowering effect of acupuncture required for managing hypertension. Further RCTs with at least 7 days, and preferably longer, follow-up periods are needed (23). In addition, there has been a lack of research that focused on the effect of acupuncture on reducing the recurrence rate of hypertensive stroke patients.

We conducted this multicenter, long follow-up RCT to evaluate the efficacy and safety of acupuncture as adjunctive therapy for ischemic stroke patients with hypertension. The efficacy of acupuncture in reducing BP was evaluated during the 12-week intervention and the 48-week follow-up period, and stroke recurrence was evaluated in one year.

## 2 Materials and methods

### 2.1 Study design

This RCT was conducted in clinics and wards of 18 centers from September 2015 to January 2020. The number of centers was increased from 5 to 18 due to the difficulty of participant enrollment. Eventually, 480 patients with first-ever stroke and hypertension who met the screening criteria were randomized into either adjunctive acupuncture or basic treatment group by a 1:1 ratio to complete the 12-week treatment and the following 48-week follow-up.

### 2.2 Participants

#### 2.2.1 Inclusion criteria

(1) First-ever ischemic stroke patients with stroke duration of 2 to 6 weeks;
(2) Aged 35 to 70 years;
(3) SBP/DBP below 160/100 mmHg using single antihypertensive medication, except for the fixed-dose combinations, for at least 2 weeks;
(4) Voluntary informed consent signed.

#### 2.2.2 Exclusion criteria

(1) Secondary hypertension;
(2) Antihypertensive medication general used other than BP control;
(3) SBP/DBP below 140/90 mmHg when taking single antihypertensive drug;
(4) Other coexisting nervous system diseases, including epilepsy and peripheral nerve injuries;
(5) Severe hematopoietic system conditions, coagulation dysfunction, and cancers;
(6) Diabetic nephropathy, severe liver and renal function impairment, severe heart or lung dysfunction, and arrhythmia;
(7) Local infection around the acupoint;
(8) Pregnant or lactating women;
(9) Recruitment into other clinical trials within 1 month.

### 2.3 Randomization, concealment, and blinding

Eligible participants were randomized into adjunctive acupuncture group or basic treatment group in a 1:1 ratio. Randomization sequence was generated centrally at each center using the minimization procedure with a minimization methods (24, 25). The allocation concealment was described in the trial protocol (26). Treatment allocation was masked from center investigators, participants, and outcome assessors, with the exception of the acupuncturists.

### 2.4 Interventions

#### 2.4.1 Basic treatment group

Participants in the basic treatment group received a pharmaceutical treatment (single antihypertensive medication), conventional treatment of ischemic stroke, and “XingNao KaiQiao” (XNKQ) acupuncture for 12 weeks. The acupoint selection and needle manipulation details were described in our trial protocol (26).

The acupoints in XNKQ therapy were bilateral Neiguan (PC6), Shuigou (DU26), hemiplegic side Sanyinjiao (SP6), Jiquan (HT1), Chize (LU5), and Weizhong (BL40). After needle penetration, acupuncture manipulations were performed until participants got a Deqi response (Supplementary Table 1), and then the needles were retained for 30 min. The acupuncture intervention lasts for 12 weeks. In the first 6 weeks, the treatment was conducted five times a week (once a day), and then, in the following 6 weeks, the treatment was performed three times a week (once a day).

#### 2.4.2 Adjunctive acupuncture group

Participants in the adjunctive acupuncture group received “HuoXue SanFeng” (HXSF) acupuncture plus the basic treatment for 12 weeks. The acupoint selections of HXSF acupuncture were bilateral Renying (ST9), Hegu (LI4), Quchi (LI11), Taichong (LR3), and Zusanli (ST36). The treatment sessions of adjunctive acupuncture group were consistent with basic treatment group. The needling operation detail and acupuncture manipulation are listed in Supplementary Table 1.

### 2.5 Main outcomes and measures

The primary outcome was the recurrence rate of stroke in 1 year. The secondary outcomes included 24-h ambulatory BP was assessed at baseline, 6 and 12 weeks; home BP monitoring at 1,12,24,36,48 weeks; the Traditional Chinese Medicine (TCM), syndrome score, the Short Form 36-item Health Survey (SF-36), the National Institute of Health Stroke Scale (NIHSS), the Barthel Index (BI) scale, cardiac ultrasound, carotid artery ultrasound, transcranial doppler, and lower extremity ultrasound was evaluated at baseline, 12 weeks; serum tests at baseline, 6 and 48 weeks; The safety of acupuncture was also be assessed.

### 2.6 Sample size calculation and statistical analysis

Based on China’s Ministry of Health data, the recurrence rate of stroke was 16%. In the early clinical pretest, the recurrence rate was 7%, and the difference between groups of recurrence rate was 4.5% (26). Considering a two-sided 5% significance level and 80% power (*α* = 0.05, *β* = 0.2), with a projected dropout rate of 15%, we aimed to recruit 480 patients for 240 in each group in this study.

### 2.7 Statistical analysis

Statistical analysis was conducted by professional statisticians using SAS 9.4 statistical software. The primary outcome and 24-h ambulatory blood pressure were analyzed using the modified intention-to-treat method, which included all participates who underwent randomization, had complete baseline data, and provided at least one treatment-related outcome measurement. We used the last observation carried forward method to supplement missing values. The per-protocol analysis were retained as a sensitivity analysis. Normally distributed measurement data was described as mean value ± standard deviation, and a *t* test was used for the between-group comparison. The continuous data with repeated measurements were analyzed using a mixed-effects model. The non-normal distributed measurement data was described as median value (first quartile, third quartile), non-parametric rank sum test was used to compare groups. The categorical variables and ordinal variables were described by frequency and composition ratio. The *χ^2^* test, the Fisher exact probability method and the rank sum test were used for statistical analysis. The non-normal distributed measurement data, the categorical variables and ordinal variables with repeated measurement data was analyzed using the generalized estimating equations. All *p* values were from 2-sided tests with a significance level of < 0.05. Safety analysis was performed based on the safety analysis set.

### 2.8 Ethical considerations

Ethical approval was granted by the Ethics Committee of First Teaching Hospital of Tianjin University of Traditional Chinese Medicine (Ethics Reference No: TYLL2016[K]NO.007). All the participants will be given and signed written consent form before being recruited into our study. The study was conducted in accordance with the Declaration of Helsinki and applicable data protection legislation.

## 3 Results

### 3.1 Participants and baseline characteristics

**Figure 1:**
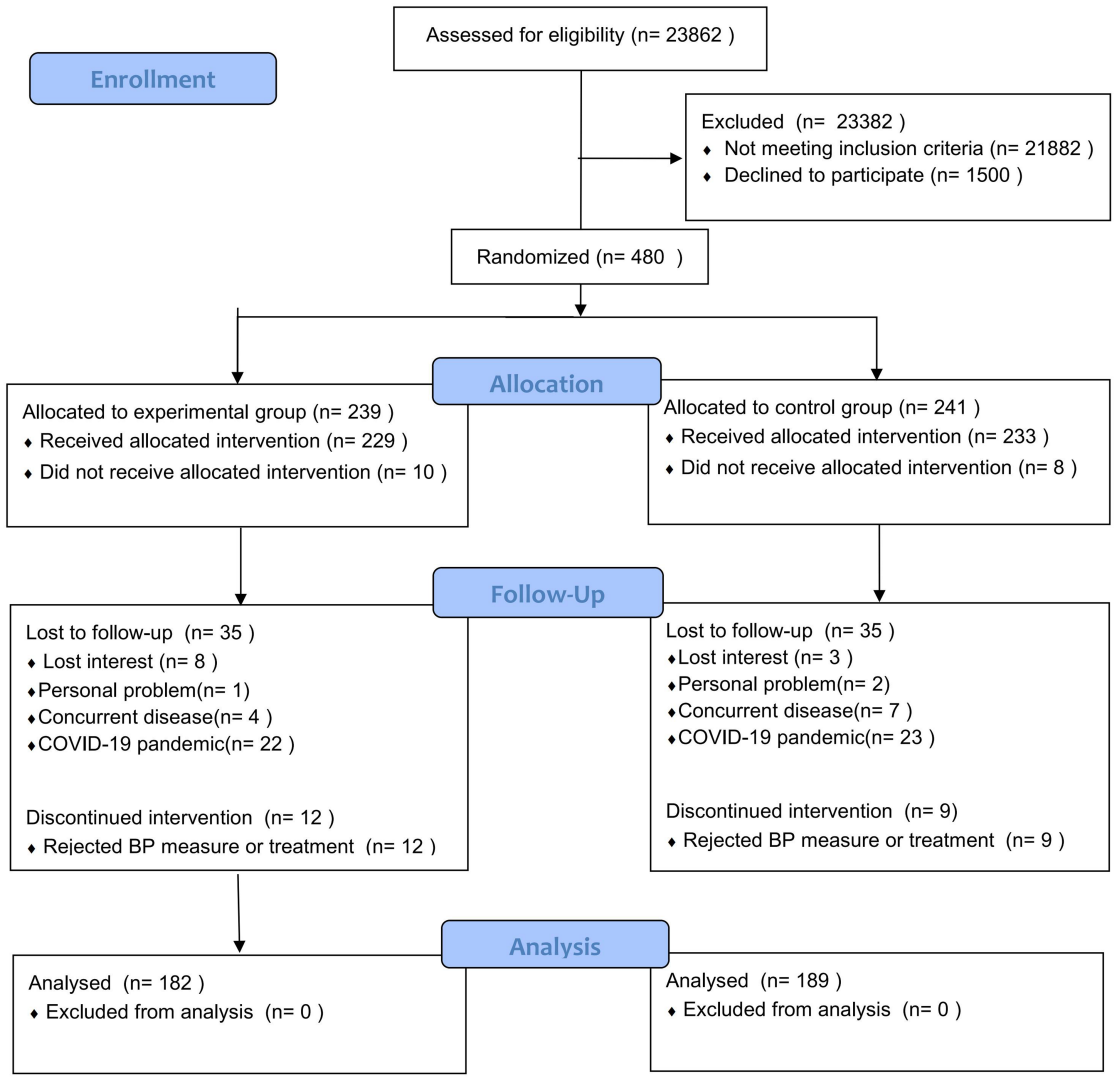
CONSORT 2010 flow diagram.

480 eligible patients were included, and 109 patients dropped out, with reasons illustrated in Figure 1. Eventually, 371 patients, including 182 in the adjunctive acupuncture group and 189 in the basic treatment group, completed the trial (Figure 1). The demographic characteristics of the 371 completed participants are presented in Table 1. The baseline information has no statistical differences (*p* > 0.05).

**Table 1:** Baseline demographic and clinical characteristics of the included participants.*

Variable	Group	EG (*n* = 182)	CG (*n* = 189)	*χ^2^/t/Z*	*p*
Sex	Male	114 (62.6)	116 (61.4)	0.06	0.80
	Female	68 (37.4)	73 (38.6)		
Age (y)	-	59.27 ± 7.58	58.69 ± 7.53	0.74	0.46
Nationality	Han nationality	175 (97.8)	186 (98.4)	-	0.72
	non-Han nationality	4 (2.2)	3 (1.6)		
Marital status	Married	175 (96.7)	178 (94.7)	0.89	0.35
	Single/Widowed/Divorced	6 (3.3)	10 (5.3)		
Educational level	<High school	106 (58.2)	117 (61.9)	1.23	0.75
	Junior college	35 (19.3)	30 (15.9)		
	>Bachelor	11 (6.0)	14 (7.4)		
	Uknown	30 (16.5)	28 (14.8)		
Height (cm)	-	168.00 ± 8.06	167.20 ± 7.72	0.95	0.34
Weight (kg)	-	71.19 ± 11.92	70.61 ± 12.29	0.46	0.64
BMI (kg/m^2^)	-	25.13 ± 3.16	25.14 ± 3.29	−0.03	0.98
BMI grade	Normal (<24)	72 (39.6)	68 (36.0)	0.52	0.77
	Overweight (≤24)	77 (42.3)	84 (44.4)		
	Obesity (≥28)	33 (18.1)	37 (19.6)		
Waistline (cm)	-	89.51 ± 9.93	88.68 ± 9.08	0.82	0.41
Waistline grade	Normal	75 (42.4)	91 (49.7)	1.96	0.16
	Abdominal obesity	102 (57.6)	92 (50.3)		
Salt intake	≤6	56 (30.9)	50 (26.6)	2.94	0.23
	7 ~ 12	96 (53.1)	95 (50.5)		
	>12	29 (16.0)	43 (22.9)		
Smoking history	Quit smoking within 1 year	12 (6.9)	14 (7.8)	0.78	0.85
	Not quit smoking	46 (26.3)	48 (26.7)		
	Quit smoking for more than a year	17 (9.7)	13 (7.2)		
	No smoking history	100 (57.1)	105 (58.3)		
Drinking history	No	130 (71.4)	122 (64.6)	2.01	0.16
	Yes	52 (28.6)	67 (35.4)		
Exercise days per week	<3 d	74 (40.9)	85 (45.0)	1.39	0.50
	3 ~ 5 d	42 (23.2)	47 (24.9)		
	6 ~ 7 d	65 (35.9)	57 (30.1)		
Exercise intensity	<30 min	83 (45.9)	84 (44.5)	0.17	0.92
	30 min ~ 1 h	69 (38.1)	76 (40.2)		
	>1 h	29 (16.0)	29 (15.3)		
Long-term mental stress or anxiety	No	142 (78.5)	144 (76.2)	0.27	0.60
	Yes	39 (21.5)	45 (23.8)		
NIHSS	-	3.0 (1.0,5.0)	3.0 (1.0,5.0)	0.10	0.92

*EG, experimental group; CG, control group.

### 3.2 Primary outcome

The modified intention-to-treat analysis showed stroke recurrence in 5 of 212 participants (2.4, 95% CI [0.80–5.40]) in the adjunctive acupuncture group versus 7 of 224 participants (3.1, 95% CI [1.30–6.30]) in the basic treatment group (*p* = 0.63), with no statistically significant difference (Table 2). Similar results were observed in the sensitivity analyses (Supplementary Table 2).

**Table 2:** Recurrence rate of stroke.

Time	Group	Recurrence (rate)	95% CI	*χ^2^*	*p*
1 year	EG (*n* = 212)	5 (2.4)	0.80, 5.40	0.24	0.63
	CG (*n* = 224)	7 (3.1)	1.30, 6.30		
More than 1 year	EG (*n* = 212)	9 (4.3)	2.00, 7.90	0.29	0.59
	CG (*n* = 224)	12 (5.4)	2.80, 9.20		

### 3.3 Secondary outcomes

#### 3.3.1 24-h ambulatory blood pressure

After 6-week treatment and 12-week treatment, the participants in both groups had a reduction of the BP value in 24 h SBP and DBP, daytime SBP and DBP, as well as nighttime SBP and DBP (*p* < 0.001, for all comparisons), while there were no statistical differences between groups (*p* > 0.05, for all comparisons) (Table 3). Similar results were observed in the sensitivity analyses (Supplementary Table 3).

**Table 3:** Comparison of the 24-h ambulatory blood pressure between 2 groups and baseline.*

Outcome assessment	EG (*n* = 212)			CG (*n* = 224)			*P_group_*	*P_time_*
	Baseline	Week 6	Week 12	Baseline	Week 6	Week 12		
24-h ambulatory blood pressure								
SBP	137 ± 15	132 ± 16	130 ± 16	136 ± 14	132 ± 13	132 ± 14	0.63	**<0.001**
DBP	81 ± 11	78 ± 11	77 ± 10	80 ± 10	78 ± 10	78 ± 10	0.95	**<0.001**
Pulse	63 ± 21	61 ± 22	61 ± 22	59 ± 16	60 ± 19	60 ± 20	0.32	0.93
Daytime								
SBP	138 ± 16	134 ± 17	133 ± 15	137 ± 15	134 ± 14	134 ± 14	0.87	**<0.001**
DBP	82 ± 12	79 ± 11	79 ± 11	81 ± 10	79 ± 10	80 ± 11	0.84	**<0.001**
Pulse	63 ± 22	61 ± 22	62 ± 22	60 ± 17	61 ± 19	61 ± 20	0.35	0.93
Night-time								
SBP	134 ± 17	128 ± 18	125 ± 17	133 ± 6	129 ± 16	126 ± 16	0.82	**<0.001**
DBP	78 ± 12	74 ± 11	73 ± 10	77 ± 11	75 ± 12	74 ± 11	0.88	**<0.001**
Pulse	62 ± 21	60 ± 20	60 ± 21	59 ± 6	59 ± 18	58 ± 20	0.26	0.33

*P_group_, the *p*-value for between-group comparisons; P_time_, the *p*-value for the time effect.

The bold values mean *p* < 0.05.

After 6 and 12-week treatment, the BP load in daytime and nighttime was significantly lower in both two groups (*p* < 0.001, for all comparisons) while there were no statistical differences between groups (*p* > 0.05, for all comparisons). No statistical differences between groups were found in the morning BP surge variety follow treatment time (*p* > 0.05). For the BP circadian rhythm, there is a higher participant proportion of dipper DBP pattern in the adjunctive acupuncture group compared with the basic treatment group (*p* = 0.01), while no statistical differences were observed in participant ratio of the dipper SBP (*p* > 0.05).

#### 3.3.2 Home blood pressure monitoring

The results showed that the SBP, DBP, and pulse pressure differences were reduced in BP value over time (*p* < 0.001, for all comparisons). No significant difference was found in BP value between groups (Table 4; Supplementary Figure 1).

**Table 4:** Comparison of the home blood pressure monitoring (mmHg) between 2 groups.*

Outcome assessments	EG (*n* = 182)					CG (*n* = 189)					P_group_	P _time_	P_group × time_
	Treatment		Follow-up			Treatment		Follow-up					
	Week 1	Week 12	Week 24	Week 36	Week 48	Week 1	Week 12	Week 24	Week 36	Week 48			
Morning													
SBP	144.11 ± 12.04	139.91 ± 10.16	138.69 ± 10.01	138.09 ± 11.09	137.70 ± 13.16	142.26 ± 11.09	138.46 ± 9.95	137.08 ± 9.18	136.37 ± 9.07	135.85 ± 9.11	0.08	**<0.001**	0.98
DBP	88.11 ± 9.23	85.75 ± 7.90	85.13 ± 7.59	84.73 ± 7.40	84.4 ± 7.24	87.42 ± 7.95	85.71 ± 7.20	85.02 ± 6.62	84.55 ± 6.33	84.19 ± 6.19	0.74	**<0.001**	0.48
Pulse	56.00 ± 10.88	54.16 ± 10.33	53.55 ± 10.48	53.37 ± 11.76	53.30 ± 13.76	54.84 ± 10.15	52.74 ± 9.93	52.06 ± 9.56	51.82 ± 9.53	51.66 ± 9.53	0.15	**<0.001**	0.97
9–11 a.m.													
SBP	140.42 ± 11.05	137.64 ± 9.45	136.37 ± 8.59	135.54 ± 8.21	134.91 ± 8.12	139.50 ± 11.39	135.51 ± 9.14	134.61 ± 8.52	134.02 ± 8.42	133.60 ± 8.38	0.08	**<0.001**	0.41
DBP	85.29 ± 8.77	83.9 ± 7.47	83.43 ± 7.08	83.04 ± 6.89	82.94 ± 7.18	85.2 ± 7.78	84.19 ± 9.12	83.42 ± 7.15	83.03 ± 6.59	82.78 ± 6.37	0.99	**<0.001**	0.86
Pulse	55.13 ± 10.32	53.74 ± 9.17	52.94 ± 8.71	52.50 ± 8.44	51.97 ± 8.78	54.30 ± 10.13	51.32 ± 10.90	51.19 ± 9.31	50.99 ± 8.97	50.83 ± 8.83	0.09	**<0.001**	0.07
3–5 pm													
SBP	141.05 ± 11.33	137.59 ± 9.14	136.59 ± 8.37	135.89 ± 8.00	135.3 ± 7.97	139.93 ± 10.18	136.5 ± 8.73	135.45 ± 7.84	135.05 ± 7.60	134.65 ± 7.64	0.24	**<0.001**	0.92
DBP	86.38 ± 8.61	84.27 ± 7.28	83.89 ± 6.92	83.58 ± 6.70	83.32 ± 6.67	85.63 ± 7.62	84.35 ± 6.67	83.85 ± 6.25	83.50 ± 6.01	83.29 ± 5.92	0.81	**<0.001**	0.12
Pulse	54.68 ± 10.36	53.32 ± 8.77	52.70 ± 8.45	52.31 ± 8.34	51.99 ± 8.29	54.30 ± 9.89	52.15 ± 8.72	51.60 ± 8.26	51.56 ± 8.17	51.36 ± 8.25	0.35	**<0.001**	0.52
Before sleeping													
SBP	139.82 ± 11.35	137.25 ± 13.49	135.92 ± 10.59	135.31 ± 10.22	134.69 ± 10.15	139.19 ± 10.38	135.95 ± 8.91	134.92 ± 8.16	134.43 ± 7.93	134.10 ± 7.99	0.35	**<0.001**	0.86
DBP	85.55 ± 8.48	83.67 ± 7.45	83.20 ± 6.94	82.92 ± 6.66	82.67 ± 6.57	85.22 ± 7.97	83.75 ± 7.02	83.26 ± 6.46	82.95 ± 6.19	82.74 ± 6.09	0.98	**<0.001**	0.74
Pulse	54.28 ± 10.88	53.58 ± 12.15	52.71 ± 9.84	52.38 ± 9.64	52.02 ± 9.52	53.97 ± 10	52.20 ± 8.40	51.67 ± 8.28	51.47 ± 8.21	51.37 ± 8.25	0.35	**<0.001**	0.53

*P_group_, the *p*-value for between-group comparisons; P_time_, the *p*-value for the time effect.

The bold values mean *p* < 0.05.

#### 3.3.3 Clinical scales

Clinical scale assessments were performed after 6-week and 12-week treatment, using TCM syndrome Scale, SF-36, NIHSS, and the BI (Supplementary Table 4). There were no statistical differences between groups in TCM syndrome scores, NIHSS, and BI between groups. Compared with the baseline, the score of each sub-item in SF-36 in each group increased after 12-week treatment, with statistical differences between baseline in physical functioning, role-physical, pain, general health, vitality, social functioning, role-emotional, and mental health (*p* < 0.001). There were no statistical differences between groups in pain, general health, vitality, role-emotional, and mental health, and control group showed higher scores in physical functioning (*p* = 0.01), role-physical (*p* = 0.01), and social functioning (*p* = 0.01).

#### 3.3.4 Ultrasound examination

Ultrasound examinations were conducted after 12-week treatment (Supplementary Table 4). For the outcome of cardiac ultrasound, no statistical differences were found in left ventricular ejection fraction, Left atrial diameter, and the percentage of participants with mitral valve E/A ≤ 1 between groups (*p* > 0.05).

For the outcome of carotid artery ultrasound, no statistical differences were found in the between-group comparisons of the internal diameter of carotid artery vessel, peak carotid vascular velocity, and carotid artery vascular resistance indices in bilateral common carotid artery, left internal carotid artery, and right internal carotid artery (RICA) (*p* > 0.05 for all comparisons), except for the carotid artery vascular resistance of RICA, of which the adjunctive acupuncture group decreased with statistical difference compared with basic treatment group (*p* = 0.03 < 0.05). There was no statistical difference between groups in both sides intima-media thickness (left side: *p* = 0.76 > 0.05; right side: *p* = 0.75 > 0.05).

As for the transcranial doppler examination after 12-week treatment, the blood flow velocity of left vertebral artery was lower than basic treatment group with statistical difference (*p* = 0.02), the blood flow velocity of the bilateral middle cerebral artery, bilateral anterior cerebral artery, bilateral posterior cerebral artery, right vertebral artery, basilar artery did not show significant statistical difference.

#### 3.3.5 Serum test

There were no significant changes between groups in the serum level of Copeptin, endothelin-1, soluble CD40, and nitric oxide. (Copeptin, *p* = 0.32; endothelin-1, *p* = 0.65; CD40, *p* = 0.78; nitric oxide, *p* = 0.29) (Supplementary Table 5).

#### 3.3.6 Antihypertensive medication

For the antihypertensive medication adjustment, compared with experimental group (10.4, 14.8, 18.1%), the control group (2.6, 2.6, 5.8%) showed a lower withdrawal in antihypertensive medication during the first 6 weeks, week 7 to week 12, and after 12 weeks of treatment (Supplementary Table 6).

#### 3.3.7 Adverse events

There were no statistical difference between groups (*p* = 0.35). After 12-week treatment and 36-week follow-up, 4 participants (1.66%) had adverse events in the basic treatment group, and 7 (2.93%) occurred in the adjunctive acupuncture group (Supplementary Tables 7, 8).

## 4 Discussion

To our knowledge, our study was the largest multicenter RCT to evaluate the long-term efficacy and safety of adjunctive acupuncture for ischemic stroke patients with hypertension. Compared to the basic treatment containing XNKQ acupuncture, adjunctive acupuncture therapy did not have a superior effect on reducing stroke recurrence in the 1-year follow-up. No group differences in reducing BP were seen. However, more participants in the adjunctive acupuncture group decreased the antihypertensive medication use during the first 6 weeks, week 7 to week 12, and after 12 weeks of treatment. In addition, adjunctive acupuncture therapy was more effective in increasing the dipper DBP proportion and improving RICA, and SF-36 for ischemic stroke patients.

Regarding the primary outcome, the modified intention-to-treat analysis showed stroke recurrence in 5 of 212 participants (2.4, 95% CI [0.80–5.40]) in the adjunctive acupuncture group versus 7 of 224 participants (3.1, 95% CI [1.30–6.30]) in the basic treatment group (*p* = 0.63), with no statistically significant difference. The relatively low event rate limited our statistical power despite the large sample size. This highlights the challenge of evaluating stroke recurrence in a stable post-stroke population receiving comprehensive medical management, particularly when both groups received active acupuncture interventions. Several factors may have contributed to this null finding. First, the one-year follow-up period, whilst adequate for assessing short- to medium-term outcomes, may have been insufficient to capture the full impact of acupuncture on stroke recurrence. Cerebrovascular events often manifest over longer time horizons. Second, our sample size was adequately powered for the anticipated effect size based on previous literature. However, the unexpectedly low event rate in both groups suggests that the trial may have been underpowered to detect clinically meaningful differences in stroke recurrence. Third, all participants received comprehensive medical management, including optimal antihypertensive therapy and secondary prevention strategies. This likely contributed to the overall low recurrence rate, potentially masking any incremental benefit of adjunctive acupuncture. This study carried out the longest follow-up on the efficacy of acupuncture in lowering BP. Previous clinical studies have suggested the short-term anti-hypertension effect of acupuncture (21, 22, 27–29). In this study, systolic and diastolic BP in the morning, 24 h, daytime, and night time were reduced after treatment in both groups. Moreover, the effects of the BP reduction persisted in both groups through the 36-week follow-up period after acupuncture intervention. It might be related to the potential antihypertension effect of the XNKQ acupuncture therapy, which is included in the basic treatment. The acupoints in XNKQ acupuncture therapy, such as SP6, were also found in the previous antihypertensive acupuncture therapy (30). Different from our results, a previous study (21) showed that the antihypertensive effect would disappear after cessation of acupuncture treatment, and BP would return to the pre-acupuncture level. The inconsistency might be related to the higher frequency of acupuncture and the longer duration of treatment in our study. Our previous study (31) also suggested that manipulation parameters and acupoint selection could affect the antihypertensive effect of acupuncture.

BP circadian rhythm is highly associated with cardiovascular and renal homeostasis, the disruption of which could contribute to adverse cardiovascular events (32). The dipper pattern of BP circadian rhythm refers to the fact that nighttime BP values are lower than daytime BP values by 10–20%, and the absence of a dipper pattern, non-dipper, is considered abnormal (33–35). The dipper pattern can independently predict mortality and cardiovascular events in hypertensive patients (36). Non-dipper is a common pattern of hypertension and is associated with increased cardiovascular disease risk (37–39). In our study, adjunctive acupuncture therapy increased the proportion of the participants with dipper DBP, thereby improving the BP circadian rhythm. Yancui Sun et al. also suggested that arterial stiffness and endothelial dysfunction were correlated with the abnormal circadian rhythm of BP (40). In our study, the proportion of participants with dipper DBP pattern increased from 28.4 to 45.6% in the adjunctive acupuncture group versus 28.7 to 31.8% in the basic treatment group at 48 weeks (*p* = 0.01). This represents clinically meaningful improvement, as restoration of normal circadian rhythm has been associated with reduced cardiovascular event risk in hypertensive populations (41). The conversion from non-dipper to dipper pattern may contribute to cardiovascular protection independent of absolute blood pressure reduction.

In addition, this study also suggested that adjunctive acupuncture group had superior benefits in reducing the resistance index of RICA than basic treatment group, indicating that HXSF acupuncture can help improve carotid blood flow. The resistive index of ICA is a predictor of cardiovascular mortality and morbidity (42). Stroke patients appeared to have larger common carotid artery diameters, lower carotid flow velocities and volume, and higher resistance index than non-stroke patients independently of extracranial carotid atherosclerosis (43). Adjunctive acupuncture might improve vascular dysfunction and protect the target organs by regulating the BP rhythm. The observed reduction in RICA resistance index (*p* = 0.03), whilst modest, may have clinical significance. Altered carotid hemodynamics have been associated with adverse cerebrovascular outcomes (44), suggesting that the improvement we observed may reflect enhanced cerebral perfusion.

Moreover, adjunctive acupuncture is more effective in improving the quality of life of ischemic stroke patients with hypertension. It had superior benefits in improving the score of SF-36, the most widely used generic instrument for measuring the quality of life (45) in the domains of physical functioning, role-physical, pain, general health, vitality, social functioning, role-emotional, and mental health. The apparent discrepancy between improved SF-36 scores and unchanged NIHSS/BI scores warrants clarification. NIHSS and BI primarily assess acute stroke severity and basic activities of daily living, which may lack sensitivity for detecting subtle improvements in chronic stroke recovery. In contrast, SF-36 captures broader health-related quality of life dimensions. Our findings suggest that whilst adjunctive acupuncture may not alter gross neurological function, it may enhance patients’ perceived physical capacity and reduce activity limitations. However, we acknowledge that we did not assess whether the observed SF-36 changes reached the minimum clinically important difference, which has been estimated at 1.8–3.0 points for the Physical Component Summary in stroke patients (46, 47).

Hypertension is one of the most prevalent risk factors for stroke (2, 48). A previous randomized trial of 6,105 individuals with previous stroke or transient ischaemic attack showed annual stroke recurrence rates of 2.7% with perindopril plus indapamide treatment versus 3.8% with placebo (49). Although it may be caused by the difference in the population or basic treatment between these two studies, the first-year stroke recurrence rate in both groups in our study is lower than that of the previous study. Ming-Cheng Huang et al. also suggested that acupuncture might have a beneficial effect on reducing the risk of stroke in patients with fibromyalgia (50). The effect of reducing stroke recurrence might be related to the decreased BP in both groups, and the basic treatment containing XNKQ acupuncture’s effect on improving the level of neurotransmitters and cerebral blood flow perfusion and stroke rehabilitation (51–53). Although adjunctive acupuncture may have potential benefits in improving circadian rhythm, carotid blood flow, quality of life, and lowering the use of antihypertension medication, it did not present a superior effect on reducing stroke recurrence in the 1-year follow-up. Further clinical studies with extended follow-up periods and a control group without acupuncture intervention or sham acupuncture are recommended to further explore the efficacy of acupuncture in reducing stroke recurrence.

ST9 is the key acupoint for HXSF acupuncture, located at the pulsation of the carotid artery, the deep part of which is the carotid sinus. The stimulation of carotid sinus stimulators may engage baroreflex afferent activity to govern efferent sympathetic and parasympathetic activity, thereby controlling the BP (54). This mechanistic hypothesis provides a potential explanation for our observed improvements in BP circadian rhythm (increased dipper DBP pattern) and reduced carotid artery resistance index, as baroreceptor-mediated autonomic modulation could theoretically restore physiological BP variation and improve vascular function. A previous study suggested that acupuncture at ST9 could significantly lower the BP of spontaneously hypertensive rats and affect their hypothalamic gene expression profile (55). More participants in the adjunctive acupuncture group decreased the antihypertensive medication use during the first 6 weeks, week 7 to week 12, and after 12 weeks of treatment. It indicates that compared with basic treatment, adjunctive acupuncture therapy may have superior effects on reducing BP, which is consistent with our previous, small-sample, single-center studies on HXSF acupuncture (56, 57). However, as this clinical trial measured patient outcomes rather than direct mechanistic endpoints, we cannot definitively establish causal pathways. The associations between ST9 acupuncture and physiological improvements remain hypothesis-generating, and dedicated mechanistic studies with direct measures of autonomic function, neurohormoral markers, and vascular reactivity are needed to elucidate the biological mechanisms underlying these clinical observations.

## 5 Limitations

This study has several limitations. First, although the sample size of this study is relatively large, the dropout rate is still high. Due to the impact of Corona Virus Disease 2019, some patients could not go to the hospital to complete the intervention and follow-up. There are 109 patients who withdrew from the study, which may influence the stability of the study results. The 22.7% attrition rate (109 of 480 participants), partly due to COVID-19-related restrictions, may affect the generalizability of our findings. Although modified intention-to-treat analysis was employed to minimize attrition bias, the higher-than-anticipated loss to follow-up could potentially introduce selection bias and limit the external validity of our results, particularly for the long-term stroke recurrence outcome. Second, the basic treatment of our study includes a basic acupuncture therapy for stroke rehabilitation, XNKQ acupuncture. As XNKQ acupuncture is a common and basic treatment for stroke complications in China, it’s hard to exclude it from the basic treatment of stroke. However, this active control design reduces the ability to detect treatment differences, as both groups received interventions that may independently contribute to stroke prevention. This limitation is particularly relevant for interpreting the negative primary outcome, as we evaluated the incremental effect of HXSF over XNKQ rather than the overall efficacy of acupuncture versus no treatment. Further clinical studies with blank or placebo control are still needed to explore acupuncture efficacy in reducing stroke recurrence. Since the multiple analyzed secondary outcomes (e.g., blood pressure parameters, SF-36 subscales) were pre-specified and non-confirmatory, a unified adjustment for multiple comparisons was not performed. Thus, the results of these analyses should be interpreted with caution. Finally, although the follow-up period of this study is fairly long compared to other clinical studies of acupuncture therapy for hypertension, a further extended follow-up period is recommended to evaluate stroke recurrence.

## 6 Conclusion

Compared to basic treatment containing XNKQ acupuncture, adjunctive HXSF acupuncture did not demonstrate a superior effect on reducing stroke recurrence at 1-year follow-up. Whilst adjunctive acupuncture was associated with improvements in several secondary outcomes, including dipper diastolic blood pressure pattern, RICA resistance index, quality of life measures, and reduced antihypertensive medication use, these findings should be interpreted cautiously given the negative primary outcome and the modest magnitude of some observed effects. Further research is needed to establish the clinical significance of these secondary findings.

## Data Availability

The original contributions presented in the study are included in the article/Supplementary material, further inquiries can be directed to the corresponding author.
