# Supplementary material for: Effect of acupuncture on ischemic stroke patients with hypertension: a randomized clinical trail
Source: Front Neurol. 2026 Apr 1;17:1717706. doi: 10.3389/fneur.2026.1717706 (PMC13079042; doi:10.3389/fneur.2026.1717706)

Supplementary Material

Supplementary Table1. Acupuncture point selection, acupoint location, and acupuncture method

Supplementary Table2. Recurrence rate of stroke

Supplementary Table3. Comparison of the 24-h Ambulatory Blood Pressure Between 2 Groups and Baseline

Supplementary Table4. NIHSS, TCM syndrome Scale, BI, SF-36[P50(P25, P75)] and Results of cardiac ultrasound, carotid artery ultrasound, Transcranial Doppler examination

Supplementary Table5. Levels of CD40, ET-1, NO and Copeptin in stroke patients after acupuncture at different time points

Supplementary Table6. Analysis of adjustment of antihypertensive drugs

Supplementary Table7. Adverse Events

Supplementary Table8. Adverse Events description

Supplementary Figure1. Results of Home Blood Pressure Monitoring

**Supplementary Table1. Acupuncture point selection, acupoint location, and acupuncture method**

| **Points** | **Location** | **Acupuncture method** |
| --- | --- | --- |
| Renying  (ST9) | In the neck, 1.5 B-cun next to the thyroid cartilage, the anterior border of sternocleidomastoid, poeterior to the common carotid artery | Patients took the hypothesis, exposed to the neck, the operator touched the artery with hand and pulsated it, inserted the needle 1~1.5 B-cun vertically. Visible needle body were swinging with pulses. Using the twisting technique of the reinforcing method of the second definition from Xue-min Shi academician, namely low amplitude (torsional amplitude less than 90°), high-frequency (120–160 times/min), the operation lasting for 1 min, retained for 30 minutes |
| Hegu  (LI4) | In the back of the hand, between the first and second metacarpals, at the midpoint of the second metacarpal radially | The needle was inserted vertically from 0.8 ~ 1 B-cun, using the twisting technique of the first definition of the reducing method from Xue-min Shi, academician. The operator was facing the patient, taking the Ren and Du two veins as the center, and the direction of the tangential force of thumb twerting is centrifugal, the operation lasting for 1 min, retained for 30 minutes |
| Taichong  (LR3) | In the dorsum of the foot, between the first and second toes, at the head of the first metatarsal space |  |
| Quchi  (LI11) | In the lateral end of the cubital crease, at the midpoint of the ligature between the end point of the cubital crease and the lateral epicondyle of the humerus with the elbow bent | The needle was inserted vertically from 1 B-cun, using the twisting technique of the first definition of the reinforcing method from Xue-min Shi, academician. The operator was facing the patient, taking the Ren and Du two veins as the center, and the direction of the tangential force of thumb twerting is centripetal, the operation lasting for 1 min, retained for 30 minutes |
| Zusanli  (ST36) | In the anterior lateral of crus, 3 B-cun under Dubi (ST35), one transverse finger width from the anterior tibial border |  |

**Supplementary Table1. Acupuncture point selection, acupoint location, and acupuncture method (continued)**

| **Points** | **Location** | **Acupuncture method** |
| --- | --- | --- |
| Neiguan  (PC6) | In the volar aspect of the forearm, at the junction of Quze (PC 3) and Daling (PC7), 2 B-cun above the wrist crease in the space between tendon palmaris longus and vagina tendinis musculi flexoris carpi radialis | The needle was inserted 1~1.5 B-cun straight into the bilateral acupoints, using the combination of the reducing method of twirling lifting and thrusting needles, the operation lasts for 1–3 mins |
| Shuigou  (DU26) | In the face, at the intersection point of the upper 1/3 and middle 1/3 of the philtrum groove | Using the reducing method of “sparrow-pecking” until tears fall or appear in the eyes |
| Sanyinjiao  (SP6) | In crus inside, 3 B-cun above the upper border of the medial malleolus of the ankle at the posterior border of the tibia | At the posterior margin of the affected tibia, the needle tip was obliquely inserted at 45° from the skin and inserted 1~1.5 B-cun, using the reinforcing method of twirling, lifting, and thrusting needles, until the lower limb consecutively twitches 3 times |
| Jiquan  (HT1) | At the apex of the axilla, the site of the pulse of the axillary artery | On the affected side, according to the anatomical characteristics of Jiquan point, the Xingnao Kaiqiao acupuncture method was used to extend it 1 to 2 B-cun downward, avoiding the axillary hair, and the acupoints were selected at the muscle fat. The needle was inserted 1~1.5 B-cun using the reducing method of twirling, lifting, and thrusting needles, until the upper extremity twitches 3 times |

**Supplementary Table1. Acupuncture point selection, acupoint location, and acupuncture method (continued)**

| **Points** | **Location** | **Acupuncture method** |
| --- | --- | --- |
| Chize  (LU5) | In the cubital crease, at the depression of the bicipital muscle tendon radialis at the elbow | Took the affected side, asked the patient to bend the elbow to an internal Angle of 120°, and the operator to support the affected wrist joint. The needle was inserted 0.5~0.8 B-cun straight using the reducing method of twirling, lifting, and thrusting needles, until the needling feeling was felt from the elbow joint to the fingers; or manual extorsion 3 times |
| Weizhong  (BL40) | At the midpoint of the popliteal crease, at the midpoint between the femoral biceps tendon and the semitendinosus tendon | Took the affected side, the patient was supine, the operator held the affected side ankle joint and lifted the affected side lower limb. The needle tip was inserted 1~1.5 B-cun outward 15°，using the reducing method of twirling, lifting, and thrusting needles, until the lower extremity twitches 3 times |

1. cun: proportional bone B-cun. This method divides the height of the human body into 75 equal units. Using joints on the surface of the body as the primary landmarks, the length and width of every bodily region is measured by such proportions.

**Supplementary Table2. Recurrence rate of stroke**

| **Time** | **Group** | **Recurrence (rate)** | ***χ^2^*** | ***P*** |
| --- | --- | --- | --- | --- |
| 1 year | EG(n=182) | 4(2.2) | 0.00 | >0.999 |
|  | CG(n=189) | 5(2.6) |  |  |
| More than 1 year | EG(n=182) | 8(4.4) | 0.16 | 0.59 |
|  | CG(n=189) | 10(5.3) |  |  |

**Supplementary Table3. Comparison of the 24-h Ambulatory Blood Pressure Between 2 Groups and Baseline***

| **Outcome assessment** | **EG(n=182)** | | |  | **CG(n=189)** | | | ***P_group_*** | ***P _time_*** |
| --- | --- | --- | --- | --- | --- | --- | --- | --- | --- |
|  | **Baseline** | **Week 6** | **Week 12** |  | **Baseline** | **Week 6** | **Week 12** |  |  |
| 24-h Ambulatory Blood Pressure | | | | | | | | | |
| SBP | 137±15 | 131±16 | 130±16 |  | 137±14 | 131±13 | 131±14 | 0.64 | **< 0.001** |
| DBP | 81±11 | 77±10 | 77±10 |  | 80±10 | 77±10 | 78±11 | 0.59 | **< 0.001** |
| Pulse | 61±18 | 59±19 | 60±21 |  | 60±16 | 60±18 | 60±19 | 0.91 | 0.82 |
| Daytime |  |  |  |  |  |  |  |  |  |
| SBP | 138±16 | 132±16 | 132±15 |  | 138±15 | 132±13 | 133±13 | 0.99 | **< 0.001** |
| DBP | 82±12 | 78±10 | 78±10 |  | 81±10 | 78±10 | 79±11 | 0.75 | **<0 .001** |
| Pulse | 61±19 | 60±20 | 61±21 |  | 60±17 | 60±18 | 61±20 | 0.88 | 0.73 |
| Night-time |  |  |  |  |  |  |  |  |  |
| SBP | 134±17 | 126±17 | 125±17 |  | 133±16 | 127±16 | 125±17 | 0.64 | **< 0.001** |
| DBP | 78±13 | 73±11 | 72±10 |  | 77±11 | 74±12 | 73±11 | 0.41 | **< 0.001** |
| Pulse | 60±18 | 58±18 | 59±21 |  | 59±16 | 59±18 | 59±20 | 0.81 | 0.50 |

**Supplementary Table3. Comparison of the 24-h Ambulatory Blood Pressure Between 2 Groups and Baseline* (continued)**

| **Outcome assessment** | **EG(n=182)** | | |  | **CG(n=189)** | | | ***P_group_*** | ***P _time_*** |
| --- | --- | --- | --- | --- | --- | --- | --- | --- | --- |
|  | **Baseline** | **Week 6** | **Week 12** |  | **Baseline** | **Week 6** | **Week 12** |  |  |
| 24-h Ambulatory Blood Pressure Load | | | | | | | | | |
| Daytime |  |  |  |  |  |  |  |  |  |
| SBP | 38.00(18.00,80.80) | 25.00(10.00,56.00) | 27.50(8.20,54.90) |  | 47.50(24.00,75.00) | 27.60(11.00,57.00) | 31.00(12.00,55.20) | 0.52 | **< 0.001** |
| DBP | 24.00(6.00,55.00) | 13.00(3.00,38.00) | 11.05(0.00,36.90) |  | 22.00(6.00,55.56) | 14.00(3.80,41.00) | 15.50(4.00,37.00) | 0.80 | **< 0.001** |
| Night-time |  |  |  |  |  |  |  |  |  |
| SBP | 75.00(44.00,100.00) | 57.00(22.00,86.00) | 43.00(13.00,87.70) |  | 77.90(41.70,100.00) | 62.00(30.00,87.50) | 50.00(22.00,85.00) | 0.33 | **< 0.001** |
| DBP | 60.00(25.00,88.90) | 41.50(11.00,75.00) | 38.00(4.15,75.00) |  | 63.00(22.00,86.00) | 45.00(13.00,80.00) | 37.50(10.00,75.00) | 0.50 | **< 0.001** |
| Morning Peak Blood Pressure | | | | | | | | | |
|  | 24.00(13.20,33.80) | 22.80(13.60,31.80) | 23.80(15.20,32.40) |  | 22.80(14.10,33.40) | 21.20(12.80,32.80) | 24.00(14.60,35.60) | 0.88 | 0.90 |
| Circadian Rhythm of Blood Pressure | | | | | | | | | |
| SBP | 3.20(-1.60, 8.33) | 4.80(0.78, 9.86) | 6.32(0.80,10.14) |  | 2.92(-2.13, 8.59) | 3.01(-1.63, 8.45) | 6.82(2.04,10.92) | 0.74 | **< 0.001** |
| DBP | 5.68(0.00,10.31) | 7.14(0.00,11.76) | 7.06(1.41,12.50) |  | 5.06(-1.19,9.72) | 4.49(-1.16,10.14) | 7.45(1.45,13.16) | 0.77 | **< 0.001** |

***^*^***: P_group_: the P-value for between-group comparisons, P_time_: the P-value for the time effect; The bold values mean P＜0.05.

**Supplementary Table4. NIHSS, TCM syndrome Scale ,BI,SF-36[P50(P25, P75)] and Results of cardiac ultrasound, carotid artery ultrasound,Transcranial Doppler examination ($\bar{x}\pm s$,%)***

| **Outcome assessments** | **EG(n=182)** | |  | **CG(n=189)** | | ***P_group_*** | ***P_time_*** |
| --- | --- | --- | --- | --- | --- | --- | --- |
|  | **Baseline** | **Week 12** |  | **Baseline** | **Week 12** |  |  |
| NIHSS | 3.0(1.0,5.0) | 1.0(0.0,3.0) |  | 3.0(1.0,5.0) | 1.0(0.0,2.0) | 0.27 | **< 0.001** |
| TCM | 14.0(9.0,20.0) | 6.0(3.0,10.0) |  | 13.0(8.0,18.0) | 8.0(5.0,13.0) | 0.41 | **< 0.001** |
| BI | 80.5±21.0 | 94.1±13.3 |  | 77.9±24.1 | 92.2±14.4 | 0.11 | **< 0.001** |
| **SF-36** | | | | | | | |
| Physical functioning | 50.0(20.0, 80.0) | 90.0(70.0, 95.0) |  | 45.0(22.5, 77.5) | 80.0(52.5, 90.0) | 0.01 | **< 0.001** |
| Role-Physical | 0.0(0.0, 25.0) | 75.0(0.0,100.0) |  | 0.0(0.0, 25.0) | 12.5(0.0,100.0) | 0.01 | **< 0.001** |
| Pain | 74.0(52.0,100.0) | 100.0(74.0,100.0) |  | 74.0(60.0,100.0) | 84.0(74.0,100.0) | 0.14 | **< 0.001** |
| General Health | 45.0(40.0,55.0) | 65.0(50.0, 77.0) |  | 50.0(37.0,62.0) | 60.0(45.0, 72.0) | 0.14 | **<0 .001** |
| Vitality | 60.0(45.0,75.0) | 80.0(65.0, 85.0) |  | 65.0(50.0,80.0) | 70.0(55.0, 80.0) | 0.14 | **< 0.001** |
| Social functioning | 44.4(33.3,66.7) | 77.8(66.7, 88.9) |  | 44.4(33.3,66.7) | 66.7(55.6, 88.9) | 0.01 | **< 0.001** |
| Role-Emotional | 0.0(0.0, 66.7) | 100(33.3,100.0) |  | 0.0(0.0,100.0) | 100(0.0,100.0) | 0.50 | **< 0.001** |
| Mental Health | 64.0(48.0,80.0） | 80.0(68.0,88.0) |  | 64.0(52.0,84.0) | 72.0(56.0,84.0) | 0.14 | **< 0.001** |
| **cardiac ultrasound** | | | | | | | |
| Left ventricular ejection fraction (%) | 64±7 | 66±6 |  | 64±6 | 65±6 | 0.63 | **< 0.001** |
| Left atrial diameter(mm) | 34(31,37) | 34(32,37) |  | 35(32,37) | 35(32,37) | 0.58 | 0.92 |

**Supplementary Table4. NIHSS, TCM syndrome Scale ,BI,SF-36[P50(P25, P75)] and Results of cardiac ultrasound, carotid artery ultrasound,Transcranial Doppler examination ($\bar{x}\pm s$,%)* (continued)**

| **Outcome assessments** | **EG(n=182)** | | | |  | **CG(n=189)** | | ***P_group_*** | ***P_time_*** |
| --- | --- | --- | --- | --- | --- | --- | --- | --- | --- |
|  | **Baseline** | | **Week 12** | |  | **Baseline** | **Week 12** |  |  |
| Left ventricular diameter (mm) | 47(43,50) | | | 46(44,49) |  | 46(42,49) | 46(43,49) | 0.28 | 0.94 |
| Mitral valve E/A | 112(80.0) | 113(80.7) | | |  | 108(77.7) | 112(80.6) | 0.77 | 0.47 |
| **carotid artery ultrasound** | | | | | | | | | |
| **internal diameter of carotid artery vessel** | | | | | | | | | |
| RCCA | 6.8(6.1,7.3) | 6.8(6.2,7.3) | | |  | 6.8(6.2,7.2) | 6.7(6.2,7.1) | 0.54 | 0.14 |
| LCCA | 6.7(6.0,7.2) | 6.7(6.1,7.2) | | |  | 6.6(6.1,7.1) | 6.6(6.1,7.0) | 0.88 | **0.03** |
| RICA | 5.0(4.4,5.5) | 5.0(4.5,5.6) | | |  | 5.1(4.5,5.6) | 5.1(4.5,5.6) | 0.12 | 0.54 |
| LICA | 5.0(4.5,5.5) | 5.1(4.7,5.6) | | |  | 5.1(4.7,5.6) | 5.0(4.7,5.7) | 0.24 | **0.01** |
| **peak carotid vascular velocity** | | | | | | | | | |
| RCCA | 60.0(48.0,75.0) | 63.0(53.0,74.0) | | |  | 62.0(50.5,76.5) | 67.0(53.0,78.0) | 0.47 | **0.01** |
| LCCA | 68.0(55.0,82.0) | 70.0(60.0,81.0) | | |  | 70.0(53.0,81.0) | 69.0(56.0,81.0) | 0.82 | 0.07 |
| RICA | 56.0(45.9,65.0) | 56.0(45.0,66.7) | | |  | 55.0(43.0,67.0) | 53.0(45.0,67.0) | 0.27 | 0.15 |
| LICA | 57.0(46.0,69.0) | 57.0(45.0,69.0) | | |  | 57.0(43.0,70.0) | 58.0(47.0,70.0) | 0.37 | 0.12 |
| **carotid artery vascular resistance indices** | | | | | | | | | |
| RCCA | 0.73±0.10 | 0.73±0.07 | | |  | 0.74±0.17 | 0.73±0.11 | 0.63 | 0.87 |
| LCCA | 0.72±0.10 | 0.72±0.07 | | |  | 0.74±0.16 | 0.72±0.11 | 0.52 | 0.80 |
| RICA | 0.62±0.08 | 0.61±0.07 | | |  | 0.64±0.13 | 0.63±0.11 | **0.03** | 0.80 |
| LICA | 0.61±0.09 | 0.61±0.07 | | |  | 0.63±0.16 | 0.63±0.13 | 0.07 | 0.92 |

|  |
| --- |

|  |
| --- |

**Supplementary Table4. NIHSS, TCM syndrome Scale ,BI,SF-36[P50(P25, P75)] and Results of cardiac ultrasound, carotid artery ultrasound,Transcranial Doppler examination ($\bar{x}\pm s$,%)* (continued)**

| **Outcome assessments** | **EG(n=182)** | |  | | **CG(n=189)** | | | ***P_group_*** | ***P_time_*** |
| --- | --- | --- | --- | --- | --- | --- | --- | --- | --- |
|  | **Baseline** | **Week 12** |  | | **Baseline** | **Week 12** | |  |  |
| **intima‐media complex thickness of artery (IMT)** | | | | | | | | | |
| Left side | 116(80.6) | 125(85.0) |  | 114(78.6) | | 127(84.7) | | 0.76 | **0.03** |
| Right side | 121(82.9) | 121(82.9) |  | 117(81.3) | | 123(82.0) | | 0.75 | 0.87 |
| **Transcranial Doppler** | | | | | | | | | |
| RMCA | 58.0(46.0,83.0) | 60.0(49.0,84.0) |  | | 55.0(45.0,72.0) | | 59.5(47.0,74.0) | 0.19 | 0.15 |
| LMCA | 57.0(46.0,80.0) | 59.0(47.0,77.0) |  | | 56.5(44.0,81.0) | | 60.0(48.0,75.0) | 0.19 | 0.90 |
| LACA | 50.0(42.0,61.0) | 49.0(42.0,60.9) |  | | 50.4(39.0,61.0) | | 51.3(41.0,66.0) | 0.23 | 0.39 |
| RPCA | 35.0(26.0,42.0) | 36.0(30.0,42.0) |  | | 36.0(29.0,42.0) | | 38.5(33.0,46.0) | 0.76 | **0.02** |
| LPCA | 34.5(25.0,40.0) | 35.0(29.0,41.0) |  | | 35.0(28.0,39.0) | | 35.0(31.0,43.0) | 0.40 | **0.02** |
| RVA | 33.0(27.0,41.0) | 33.0(26.5,38.0) |  | | 30.1(25.0,40.1) | | 34.0(29.0,43.0) | 0.47 | 0.44 |
| LVA | 31.0(24.0,38.0) | 31.0(25.0,38.0) |  | | 32.0(26.0,41.0) | | 34.0(26.0,47.0) | **0.02** | 0.53 |
| BA | 33.0(26.0,42.0) | 34.0(25.5,42.0) |  | | 34.0(25.9,45.0) | | 34.0(27.0,49.8) | 0.10 | 0.19 |

^*^RCCA: right common carotid artery; LCCA: left common carotid artery; RICA: right internal carotid artery; LICA: left internal carotid artery;RMCA: right middle cerebral artery; LMCA: left middle cerebral artery; RACA: right anterior cerebral artery; LACA: left anterior cerebral artery; RPCA: right posterior cerebral artery; LPCA: left posterior cerebral artery; RVA: right vertebral artery; LVA: left vertebral artery; BA: basilar artery.

***^*^***: P_group_: the P-value for between-group comparisons, P_time_: the P-value for the time effect; The bold values mean P＜0.05.

**Supplementary Table5. Levels of CD40, Endothelin-1, Nitric Oxide and Copeptin in stroke patients after acupuncture at different time points *P50* (*P25*, *P75*)μmol/L***

| **Indicators** | **Group** | **Baseline** | **Week 6** | **Week 24** | ***F_group_*** | ***P_group_*** | ***F_time_*** | ***P_time_*** | ***F_group×time_*** | ***P_group×time_*** |
| --- | --- | --- | --- | --- | --- | --- | --- | --- | --- | --- |
| CD40 | EG | 677.07(426.22, 1399.50) | 505.03(242.91,1153.99) | 498.42(239.33,1404.16) | 0.08 | 0.78 | 0.38 | 0.68 | 0.28 | 0.76 |
|  | CG | 801.69(457.23, 1280.17) | 624.26(300.23,1150.67) | 485.98(247.79,1350.68) |  |  |  |  |  |  |
| Endothelin-1 | EG | 43.08(27.20, 62.03) | 37.23(22.43,53.96) | 35.42(22.49,54.84) | 0.21 | 0.65 | 3.94 | **0.02** | 0.08 | 0.02 |
|  | CG | 43.18(26.64, 58.39) | 31.04(22.96,49.84) | 33.74(23.04,55.72) |  |  |  |  |  |  |
| Nitric Oxide | EG | 88.89(45.95, 130.00) | 78.79(46.59,106.67) | 69.07(46.38,120.50) | 1.12 | 0.29 | 0.28 | 0.76 | 2.20 | 0.11 |
|  | CG | 76.67(47.03, 140.00) | 81.08(50.02,125.35) | 88.02(43.37,136.75) |  |  |  |  |  |  |
| Copeptin | EG | 35.70(21.92, 55.01) | 35.93(23.19,54.80) | 26.17(21.62,37.74) | 0.99 | 0.32 | 3.76 | **0.02** | 1.49 | 0.23 |
|  | CG | 29.68(21.23, 52.41 | 36.56(23.81,55.46) | 29.95(24.43,44.51) |  |  |  |  |  |  |

***^*^***: P_group_: the P-value for between-group comparisons, P_time_: the P-value for the time effect; The bold values mean P＜0.05.

**Supplementary Table6. Analysis of adjustment of antihypertensive drugs**

| **Time** | **Within 6 weeks of treatment** | |  | **Treatment for 7 to 12 weeks** | |  | **After 12 weeks of treatment** | |
| --- | --- | --- | --- | --- | --- | --- | --- | --- |
|  | **EG** | **CG** |  | **EG** | **CG** |  | **EG** | **CG** |
| **Analysis of the increase of antihypertensive drugs in stroke patients n(%)** | | | | | |  |  | |
| 3 days to 1 week | 0(0.0) | 3(15.8) |  | 1(5.3) | 0(0.0) |  | 1(4.5) | 0(0.0) |
| 1 to 2 weeks | 3(18.8) | 1(5.2) |  | 2(10.5) | 0(0.0) |  | 1(4.5) | 1(4.0) |
| 2 weeks to 1 month | 1(6.2) | 3(15.8) |  | 3(15.8) | 0(0.0) |  | 4(18.2) | 1(4.0) |
| more than 1 month | 12(75.0) | 12(63.2) |  | 13(68.4) | 19(100.0) |  | 16(72.8) | 23(92.0) |
| total | 16(100.0) | 19(100.0) |  | 19(100.0) | 19(100.0) |  | 22(100.0) | 25(100.0) |
| **Analysis of reduction of antihypertensive drugs after increasing drug dosage in stroke patients n(%)** | | | | | | | | |
| 3 days to 1 week | 1(33.3) | 0(0.0) |  | 1(33.3) | 0(0.0) |  | 0(0.0) | 0(0.0) |
| 1 to 2 weeks | 1(33.3) | 0(0.0) |  | 1(33.3) | 0(0.0) |  | 1(20.0) | 0(0.0) |
| 2 weeks to 1 month | 0(0.0) | 1(50.0) |  | 0(0.0) | 0(0.0) |  | 1(20.0) | 1(16.7) |
| more than 1 month | 1(33.3) | 1(50.0) |  | 1(33.3) | 1(100.0) |  | 3(60.0) | 5(83.3) |

**Supplementary Table6. Analysis of adjustment of antihypertensive drugs(continued)**

| **Time** | **Within 6 weeks of treatment** | |  | **Treatment for 7 to 12 weeks** | |  | | **After 12 weeks of treatment** | | |  |
| --- | --- | --- | --- | --- | --- | --- | --- | --- | --- | --- | --- |
|  | **EG** | **CG** |  | **EG** | **CG** |  | | **EG** | **CG** | |  |
| total | 3(100.0) | 2(100.0) |  | 3(100.0) | 1(100.0) |  | 5(100.0) | | | 6(100.0) | |
| **Analysis of withdrawal of antihypertensive drugs in stroke patients n(%)** | | | | | | | | | | |  |
| 3 days to 1 week | 4(21.1) | 3(60.0) |  | 0(0.0) | 1(20.0) |  | 3(9.1) | | | 1(9.1) | |
| 1 to 2 weeks | 4(21.1) | 0(0.0) |  | 2(7.4) | 1(20.0) |  | 3(9.1) | | | 1(9.1) | |
| 2 weeks to 1 month | 7(36.7) | 1(20.0) |  | 3(11.1) | 2(40.0) |  | 3(9.1) | | | 3(27.3) | |
| more than 1 month | 4(21.1) | 1(20.0) |  | 22(81.5) | 1(20.0) |  | 24(72.7) | | | 6(54.5) | |
| total | 19(100.0) | 5(100.0) |  | 27(100.0) | 5(100.0) |  | 33(100.0) | | | 11(100.0) | |
| **Analysis of increased and resumed use of antihypertensive drugs after withdrawal in stroke patients n(%)** | | | | | | | | | | |  |
| 3 days to 1 week | 1(50.0) | 1(100.0) |  | 1(50.0) | 1(50.0) |  | 0(0.0) | | | 0(0.0) | |
| 1 to 2 weeks | 0(0.0) | 0(0.0) |  | 0(0.0) | 1(50.0) |  | 1(12.5) | | | 1(33.3) | |
| 2 weeks to 1 month | 1(50.0) | 0(0.0) |  | 1(50.0) | 0(0.0) |  | 4(50.0) | | | 0(0.0) | |
| more than 1 month | 0(0.0) | 0(0.0) |  | 0(0.0) | 0(0.0) |  | 3(37.5) | | | 2(66.7) | |
| total | 2(100.0) | 1(100.0) |  | 2(100.0) | 2(100.0) |  | 8(100.0) | | | 3(100.0) | |

**Supplementary Table7. Adverse Events (n, %)**

| Adverse events **Degree** | **EG (n=241)** | **CG (n=239)** | ***χ^2^*** | ***P*** |
| --- | --- | --- | --- | --- |
| **Total a**dverse events | 4 (1.66%) | 7 (2.93%) | 0.86 | 0.35 |
| **Mild** | 2(0.83%) | 2(0.84%) |  |  |
| **Moderate** | 1(0.41%) | 3(1.25%) |  |  |
| **Serious** | 1(0.41%) | 2(0.84%) |  |  |

**Supplementary Table8. Adverse Events description**

| **Clinical Sites** | **Randomization number** | **Adverse events description** | **Outcome (recover/death/relieved)** | **Whether associated with acupuncture** |
| --- | --- | --- | --- | --- |
| Changsha Traditional Chinese Medicine Hospital | CS009 | Recurrence of hemorrhagic cerebral infarction. |  |  |
| The First Affiliated Hospital of Hunan University of Chinese Medicine | HN012 | Cold, fever, cough, left back pain,. | The cold was recover, and the left back pain was relieved. | Definitely unrelated |
| Qingdao Traditional Chinese Medicine Hospital | QD019 | Pneumonia. |  |  |
| The First Teaching Hospital of Tianjin University of Traditional Chinese Medicine | TJ002 | Fever consideration for infection. |  |  |

**Supplementary Table8. Adverse Events description (continued)**

| **Clinical Sites** | **Randomization number** | **Adverse events description** | **Outcome (recover/death/relieved)** | **Whether associated with acupuncture** | |
| --- | --- | --- | --- | --- | --- |
| The First Teaching Hospital of Tianjin University of Traditional Chinese Medicine | TJ063 | Recurrence of cerebral infarction. | Sequela. | Definitely unrelated | |
| The First Teaching Hospital of Tianjin University of Traditional Chinese Medicine | TJ121 | Upper respiratory tract infection |  | Definitely unrelated |  |
| The First Teaching Hospital of Tianjin University of Traditional Chinese Medicine | TJ176 | Death from sudden myocardial infarction. | Death. | Definitely unrelated |  |

**Supplementary Table8. Adverse Events description (continued)**

| **Clinical Sites** | **Randomization number** | **Adverse events description** | **Outcome (recover/death/relieved)** | **Whether associated with acupuncture** | |
| --- | --- | --- | --- | --- | --- |
| Changchun Traditional Chinese Medicine Hospital | CC001 | Recurrence of cerebral infarction. | Relieved. | Perhaps unrelated |  |
| The First Teaching Hospital of Tianjin University of Traditional Chinese Medicine | TJ004 | Urinary blood and pain for 4 days. | Relieved. | Definitely unrelated |  |
| The First Teaching Hospital of Tianjin University of Traditional Chinese Medicine | TJ043 | Right metacarpal bone fracture caused by slipping and falling on a snowy road. |  |  |  |

**Supplementary Table8. Adverse Events description (continued)**

| **Clinical Sites** | **Randomization number** | | **Adverse events description** | | **Outcome (recover/death/relieved)** | **Whether associated with acupuncture** |
| --- | --- | --- | --- | --- | --- | --- |
| The First Teaching Hospital of Tianjin University of Traditional Chinese Medicine | | TJ162 | | Death due to cerebral hemorrhage. | Death. |  |

**Supplementary Figure1. Results of Home Blood Pressure Monitoring**


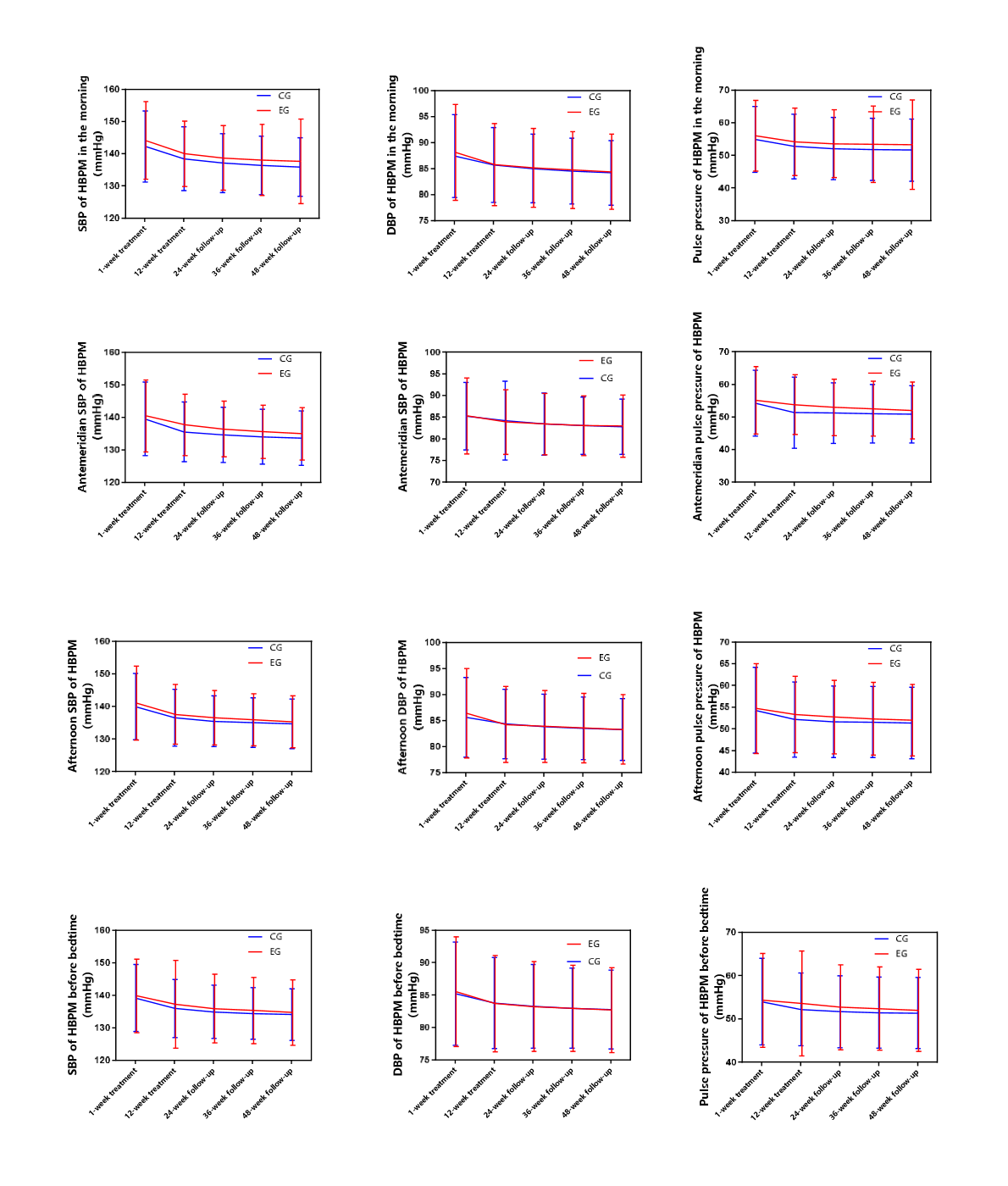

Supplement: Supplementary file 1 [file Supplementary_file_1.docx]
